# Authors’ academic but not personal expertise or gender affect message credibility in science communication

**DOI:** 10.1038/s41598-026-55148-x

**Published:** 2026-06-10

**Authors:** Annalena Ulsperger, Lara Pfannenschwarz, Clara Schetla, Karen Poletilo, Lina Strecker, Marlene Egner, Joachim Kimmerle

**Affiliations:** 1 Leibniz Institut für Wissensmedien (IWM), Tübingen, Germany; 2 University of Tübingen, Tübingen, Germany

**Keywords:** Credibility, Expertise, Science communication, Academic expertise, Personal expertise, Psychology, Psychology, Science, technology and society

## Abstract

Evaluating message credibility is critical for understanding scientific information and informed decision making. People’s ability to assess the credibility of science communication texts is influenced by several factors. In three experiments, we examined the impact of authors’ gender as well as different types of expertise on readers’ credibility perceptions. In Experiment 1 (n = 203), we varied authors’ alleged gender and expertise. We found that a text allegedly written by a high-expertise author was rated as more credible than the same text by a low-expertise author. There was no effect of authors’ gender. Experiment 2 (n = 182) was a replication of the first study with a different sample. We did not find any effects of gender or expertise on perceived message credibility. In Experiment 3 (n = 206), we differentiated between academic and personal expertise and manipulated these types of expertise independently. The data indicated that academic expertise was a significant positive predictor of perceived message credibility, while personal expertise was not. We discuss these findings in terms of their significance for the trustworthiness of science communication and examine the relevance of differentiating the concept of expertise in this context.

**Supplementary Information:**

The online version contains supplementary material available at 10.1038/s41598-026-55148-x.

## Introduction

Science communication plays an important role in everyday information transfer and can be a relevant basis for laypeople’s decision making^1^. However, this accessibility of information also entails significant risks, such as the spread of misinformation, since the verification of the content being disseminated is removed from the control of traditional gatekeepers^2,3^. Instead, the responsibility for verifying the credibility of the offered content increasingly falls onto the consumers themselves. Therefore, it is essential that people understand current science-related topics and are able to critically assess the wide range of information available^4–6^. Since laypeople often lack the specific subject-matter knowledge to reliably judge the factual accuracy of every content they encounter, they tend to consult other factors that can be used to assess the credibility of information^5,6^. These can be characteristics of the content itself like its fluency, channel characteristics like the media modality, source characteristics like the expertise of an author, or even characteristics of the recipients^7^, as credibility is subjectively perceived by message receivers^8^. In the research presented here, we focus on the potential influence of the author characteristics expertise and gender on laypeople’s subjective perception of message credibility. The nature of expertise is of special interest in this series of three studies as modern media environments have not only diluted the general reliability of whether information provided is credible, but also on who holds appropriate authority to pass on credible information in general^9^. It is, therefore, important to reexamine the notion of expertise in the modern digital climate. For this reason, Study 3 focuses on the differentiation between different types of expertise that can be encountered in information environments today. In the following paragraphs, we first introduce the concept of message credibility and its role in science communication before we present expertise and gender as two author characteristics that may influence message credibility perceptions.

### Message credibility

Message credibility refers to people’s subjective perceptions of the integrity, reliability, and trustworthiness of a given content, such as a text or other content presentation^10,11^. The credibility of a message describes the belief of receivers that the provided information is true, approvable, and trustworthy^12,13^. This belief strongly impacts the acceptance of the information provided and its effect on the receivers’ beliefs and attitudes, with more credible content being more influential than less- and non-credible content^14–17^.

Various subfactors have been used to conceptualize and quantify the perceived credibility of a message^10^. One important facet of message credibility is its trustworthiness^18^. *Trustworthiness* means that a message is perceived to be reliable and believable, and that its recipients presume a high level of veracity of that message^19^. Other relevant dimensions of credibility include the perceived truth of and the agreement with a message. *Truth* comprises the perceived accuracy of the provided statements^20^. A*greement* reflects the overlap of a receiver’s opinion with the message, which would result in approving the message and consenting to it^21^.

### Expertise

Source expertise has been shown to influence message credibility in various contexts, like in online marketing communications^22,23^, online health information^24–26^, or political information on social media platforms^27,28^. Expertise refers to the domain-specific knowledge and ability of a source, for example the author of a text, to provide accurate information in said domain developed over time^8,29,30^. Established expertise indicators are, for example, academic qualifications and credentials, as seen in health practitioners^19,31^.

In general, the literature shows that experts are considered to be more credible and persuasive than non-experts^26,27^: For example, Eastin^24^ found that study participants were more likely to believe experts when it came to health information if they themselves had no prior knowledge of the subject. Jung et al.^25^ found similar results for participants with low prior knowledge in the field of online diet and nutrition information credibility. Zimmermann et al.^28^ showed that political news on Instagram were rated higher in credibility when higher source expertise was indicated. And Meinert and Krämer^27^ showed that decision times between two sources were decreased when expertise cues were present, with expert sources being ascribed more credibility and chosen more often.

Cognitive models explaining the underlying processing of source expertise in the context of credibility perception include the Elaboration Likelihood Model^32^ and the Unifying Framework of Credibility Assessment^33^. As a dual-processing theory, the Elaboration Likelihood Model explains the advantages in processing of established expertise cues by distinguishing between central and peripheral processing, depending on the recipient’s motivation and cognitive capacities. When motivation is high and resources are sufficient, a systematic analysis of the quality of the argument takes place (central route), whereas when resources are limited, more superficial cues are used to form a credibility judgment (peripheral route)^32^. Such peripheral processing is based on heuristics, which favor established indicators like academic qualifications^34^. The notion of varied information processing can be further disentangled for the credibility context by models like the Unifying Framework of Credibility Assessment^33^. The framework distinguishes three dynamically interacting levels of credibility assessment that influence one another bidirectionally. The construct level, as the most abstract level, concerns the fundamental conceptualization of credibility (e.g., as trustworthiness or reliability). The heuristic level encompasses general beliefs or rules of thumb such as “experts are credible” or “official sources are trustworthy”^35^. At the interaction level, concrete cues are processed, including peripheral source characteristics such as titles or institutional affiliation. Crucially, these levels do not function hierarchically but rather in a circular manner. This multi-level structure illustrates how perceived credibility is shaped by both overarching credibility concepts and concrete cues, with experiences at one level influencing the others^33^. In summary, the underlying cognitive mechanisms are based on the adaptive use of heuristics, which are activated depending on available cognitive resources, motivation, conceptual understanding, and context^32,33,35^. This enables people to make efficient and valid credibility judgments in an information-rich environment.

### Gender

Another less obvious factor influencing the credibility of a text is the gender of its author. While historically female experts have been perceived as less competent than their male counterparts in science communication^36,37^, contemporary literature indicates a more equalized perception^38–40^, or even an advantage of female experts compared to males^38,41^. For example, female sports reporters covering women’s sports were rated as more likable and credible than male reporters, especially by female study participants^42^. Bundi et al.^38^ found similar preferences by their female participants for female experts in climate change communication. Jones and Mitchell^43^ even found that female experts were rated as slightly more credible in traditionally male-dominated fields such as security policy.

Taken together, prior research suggests that expertise and gender as author characteristics may have an impact on readers’ credibility perceptions regarding science communication texts. While the expertise effect is rather established, the effect of an author’s gender is subject to change. However, empirical evidence on how these factors interact in the current science communication climate remains limited. To address this research gap, we conducted a series of three studies examining the role of the authors’ characteristics in perceived credibility of a science communication article.

## Study 1

In the first study, we investigated whether the alleged expertise and gender of an author affected the perceived credibility of a science communication text. Based on the literature review and the considerations presented above, we formulated the following hypotheses:

- H1: Scientific texts by authors with high expertise are attributed greater credibility compared to texts by authors with low expertise.
- H2: Scientific texts by female authors are attributed greater credibility compared to texts by male authors.

Furthermore, we investigated as an exploratory research question based on Bundi et al.[^38^] whether there are empirical differences based on the gender of the participants (ERQ1). We also explored whether there is an interaction effect between the variables gender and expertise (ERQ2).

### Methods

All three studies reported in this paper were preregistered on AsPredicted prior to data collection (Study 1: https://aspredicted.org/cjcj-rc3b.pdf, Study 2: https://aspredicted.org/nyrs-tm7s.pdf, Study3: https://aspredicted.org/tbdk-9mx8.pdf) and approved by the Ethics Committee of the Leibniz-Institut für Wissensmedien (LEK 2024/054). Participants provided written informed consent before and after participation. Participation was anonymous and participants were informed beforehand that they had the right to discontinue the study and withdraw their data at any time during the participation. All three studies were conducted in German. The experiments were performed in accordance with relevant guidelines and regulations.

#### Participants

We conducted Study 1 as an online experiment using SoSci Survey (www.soscisurvey.de; Version 3.6.10)^44^. In a 2 × 2 between-groups design, we varied the source factors expertise (high vs. low) and gender (male vs. female) of the alleged author of a science communication text by means of an author description.

Based on an a priori power analysis (GPower, version 3.1.9.7), we aimed to recruit at least 199 participants to achieve 80% power for detecting a small effect (f = 0.2, α = 0.05) with an ANOVA with fixed effects, special, main effects and interactions. N = 234 participants completed the study, of which 31 had to be excluded due to the predefined exclusion criteria of high prior knowledge in the topic of the text or a failed attention check. Participants were recruited via the e-mail distribution list of a German university and given the option to participate in a gift-card raffle as compensation. They had to be at least 16 years old and have at least a C1 level of proficiency in German. The demographic data of the samples of all three studies are shown in Table 1.

**Table 1:** Sample size, gender, age and educational distribution for Studies 1–3.

		Study 1	Study 2	Study 3
N_total_		203	182	206
Gender				
	Female	139 (68.5%)	70 (38.5%)	50 (24.3%)
	Male	61 (30.1%)	110 (60.5%)	154 (74.8%)
	Non-binary	3 (1.5%)	1 (0.6%)	2 (1.0%)
	Other	-	-	0 (0.0%)
	Not indicated	0 (0.0%)	1 (0.6%)	0 (0.0%)
Age (years)				
	Mean (SD)	30.8 (13.2)	43.5 (12.2)	33.1 (10.2)
	Range	18–72	19–77	18–72
Educational level				
	University degree	98 (48.3%)	86 (47.3%)	92 (44.7%)
	Technical college	2 (1.0%)	19 (10.5%)	18 (8.7%)
	High school diploma	97 (47.8%)	37 (20.3%)	58 (28.2%)
	Completed apprenticeship	5 (2.5%)	26 (14.3%)	22 (10.68%)
	Secondary school	0 (0.0%)	11 (6.0%)	12 (5.8%)
	Secondary modern school	1 (0.5%)	1 (0.6%)	3 (1.5%)

#### Procedure

First, participants filled in the informed consent form. They were told that they would be asked about their assessment of a text. Afterwards, the participants were presented with one of four texts introducing an author who allegedly had written a science communication text about natural disaster preparedness, which the participants would read afterwards. Participants were distributed equally among the four conditions (*n*_high exp., fem._ = 52, *n*_high exp., male_ = 50, *n*_low exp., fem._ = 50, *n*_low exp., male_ = 51). After ten seconds the participants could move on to the next page, where they were presented with a text concerning natural disaster preparedness. The text was the same across all four conditions and was shown for at least 15 s before participants could move on. Thereafter, they were asked to judge the credibility of the text. Participants were then asked to determine the gender of the alleged author (attention check), the expertise level of the author (manipulation check), and their prior knowledge about natural disaster preparedness. Additionally, participants were directly asked whether they perceived men, women, or both as more credible in the topic. Then the participants were asked about their demographics (gender, age, and educational background) and given the option to participate in the raffle. Finally, they were debriefed and dismissed.

#### Materials and measures

The independent variables *author expertise* and *author gender* were manipulated via the author description. Expertise was presented as either high (longstanding academic career and international consulting experience with respect to the topic) or low (no academic background, only some personal interest due to previous flood experience in the author’s hometown). Gender was indicated through title, name, and pronouns as either male or female. All four versions of these stimuli are provided in the Supplementary Material (S1).

The science communication text was introduced as describing the author’s views on how sustainable and ecological methods can provide optimal protection against natural disasters. After briefly outlining the topic, the text claimed that nature-based measures alone were sufficient to prevent natural disasters. We created the text using specific prompts in ChatGPT-4 (OpenAI; Version 4.0)^45^. See Supplementary Material for the full text (S2).

We assessed message credibility using 11 items (α = 0.94) answered on a 7-point Likert scale (1 being “not at all” and 7 being “fully applies”). The scale comprised three dimensions: trustworthiness (4 items, e.g., “I consider the statements in the text to be trustworthy.”), truth (3 items, e.g., “The text reflects things as they are.”), and agreement (4 items, e.g., “I would tell a friend about this text in an approving manner.”). The items for both the trustworthiness dimension and two of the items of the agreement dimension were adapted to the present context from Klebolte^21^. The other two items of the agreement dimension were added by the authors. The truth dimension was extracted from Mehlis^20^. The message credibility score was calculated as the mean of all items. The complete scale is provided in the Supplementary Material (S3).

The question “Does the author have expertise in the subject area?” answered on a 7-point Likert scale (1 = “not at all” and 7 = “fully applies”) was used as a manipulation check.

To measure prior knowledge, we asked the following question: “Did you already have prior knowledge in the field of natural disaster preparedness?” Participants answered on a 7-point Likert scale (1 = “no prior knowledge at all” and 7 = “extremely high prior knowledge”). Participants indicating 6 or 7 on the scale were excluded from the analysis.

As an attention check, we asked “Was the author a man or a woman?” with the options: “male”, “female”, or “I don’t remember”. Participants who did not indicate the correct answer were excluded.

To ensure that the topic of the text itself was not tied to a specific gender, we asked “Do you consider men or women to be more credible in the field of preventive natural disaster preparedness?” with the answering options “male”, “female”, or “both equally”.

### Results

We used a Welch t-test to check the manipulation. It showed that the perceived expertise differed significantly between the high and the low-expertise conditions, *t*(162.73) = -13.19, *p* < 0.001,* d* = 1.86. As the authors in the high-expertise conditions were perceived as having significantly more expertise (*M* = 5.97, *SD* = 0.96) than the authors in the low-expertise conditions (*M* = 3.51, *SD* = 1.61), the manipulation was considered as successful. Furthermore, the topic of the science communication text was successfully considered gender-neutral, since most participants answered the corresponding question with “both equally” (*n*_both_ = 172, *n*_women_ = 18, *n*_men_ = 13).

**Fig. 1:**
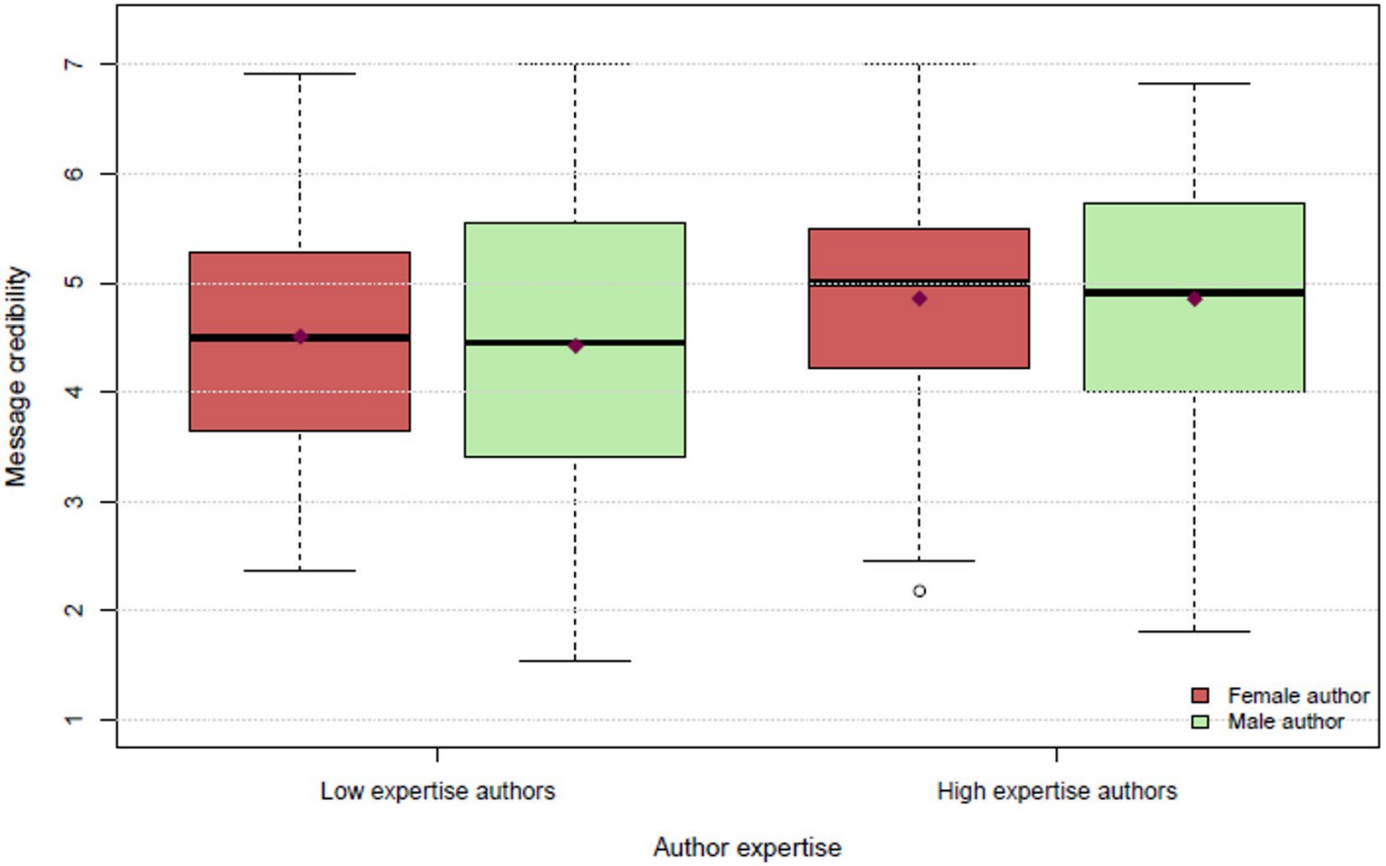
Boxplot of the perceived message credibility values for the four experimental conditions in Study 1. Mean credibility values are indicated with a lilac diamond.

To test the hypotheses that authors with high expertise (H1) and female authors (H2) would be attributed greater credibility, we conducted a two-way analysis of variance (ANOVA) with the between-groups factors expertise (high/low) and gender (female/male) of the author as independent variables and message credibility as the dependent variable. The ANOVA revealed a significant main effect of author expertise, *F*(1, 199) = 5.38, *p* = 0.021, partial *η*^*2*^ = 0.03. The results indicate that the text by authors with alleged high expertise was rated as more credible (*M* = 4.85, *SD* = 1.09) than the identical text by authors with alleged low expertise (*M* = 4.47, *SD* = 1.27), supporting H1. There was no main effect of gender, *F*(1, 199) = 0.10, *p* = 0.757, therefore rejecting H2. Regarding ERQ2, there was no significant interaction effect between the factors author expertise and author gender, *F*(1, 199) = 0.06, *p* = 0.809. The credibility values for the four conditions are depicted in Fig. 1.

To examine whether there was an empirical difference based on the gender of the participants (ERQ1), the analysis was expanded to a three-way ANOVA with participant gender as an additional between-groups factor. The main effect of author expertise remained significant, *F*(1, 193) = 5.38,* p* = 0.021, partial *η*^*2*^ = 0.03, while the main effects of author gender and participant gender were not significant, *F*s < 1.11, *p*s > 0.331. Neither of the two-way interactions, *F*s < 0.37, *p*s > 0.692, nor the three-way interaction, *F*(1, 193) = 2.75, *p* = 0.099, reached statistical significance.

### Discussion

Our findings support a positive effect of expertise on perceived message credibility in line with previous literature^24,28^. However, the observed effect was small and even the low-expertise condition received rather high credibility evaluations.

Contrary to our hypotheses and contemporary studies reporting a preference for female over male experts^38,42^, we did not find differences in credibility perceptions between male and female authors. We also observed no differences in credibility ratings based on the participants’ own gender. Instead, the results align with more recent literature suggesting a shift toward gender-independent credibility evaluations in science^38–40^, indicating a societal shift toward gender neutrality when evaluating experts. This might have been further promoted by our choice of a gender-neutral topic, which possibly minimized the salience of the author’s gender. Moreover, the lack of a gender effect might also reflect the specific characteristics of our sample. Egalitarian views and social desirability bias might be more prominent in a sample of university students.

## Study 2

To assess the generalizability of the findings of Study 1, we conducted a replication study using a sample that was more representative of the general population in terms of age and education. Since we did not observe an effect of the authors’ gender in Study 1, which is in accordance with more recent literature^38–40^, we assumed to find only a main effect of expertise in the second study:

- H1: Scientific texts allegedly written by authors with high expertise are attributed greater credibility compared to texts by authors with low expertise.

We were, again, interested in whether there were empirical differences based on the gender of the participants (ERQ1) and whether there was an interaction effect between expertise and gender of the author (ERQ2). Whether there would be a difference in credibility perceptions of the text based on the alleged gender of the author with this broader sample was also only investigated as an exploratory research question (ERQ3).

### Methods

#### Participants

The same between-groups design and exclusion criteria were used as in Study 1. However, the online experiment was hosted on Qualtrics (Qualtrics, Provo, UT). To achieve a sample more representative of the general population, participants were recruited via the online crowdsourcing platform *Clickworker https://www.clickworker.com/)*, each receiving monetary compensation of 2,14€ for completing the study. 219 participants took part in the study of which 37 had to be excluded due to the predefined exclusion criteria. In contrast to Study 1, this sample included more male than female participants, the participants were, on average, older than those in Study 1, and the educational background was more diverse with more participants indicating a completed apprenticeship, secondary school, and technical college as their highest degree. The demographic data of Study 2 is presented in Table 1.

#### Procedure

The procedure was identical to Study 1. The participants were distributed among the four conditions as follows: *n*_high exp., fem._ = 41, *n*_high exp., male_ = 45, *n*_low exp., fem._ = 47, *n*_low exp., male_ = 49.

#### Materials and measures

The same materials and measures were used as in Study 1. The internal consistency of the message credibility scale was, again, excellent with α = 0.95.

### Results

Again, a Welch t-test confirmed that the manipulation was successful, *t*(138.10) = -13.05, *p* < 0.001, *d* = 1.88. Perceived expertise differed significantly between the conditions, with authors in the high-expertise conditions being perceived as having significantly more expertise (*M* = 6.13, *SD* = 0.76) than the authors in the low-expertise conditions (*M* = 3.71, *SD* = 1.63). Participants also indicated that they again did not prefer one gender over the other in the context of preventive natural disaster preparedness (*n*_both_ = 166, *n*_women_ = 9, *n*_men_ = 7).

**Fig. 2:**
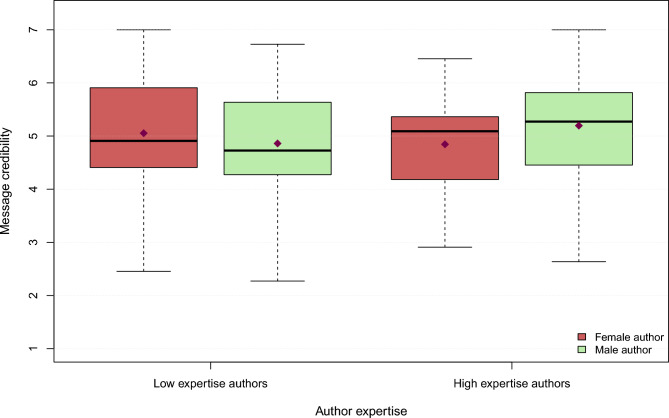
Boxplot of the perceived message credibility values for the four experimental conditions in Study 2. Mean credibility values are indicated with a lilac diamond.

As in Study 1, we used an ANOVA with the between-groups factors expertise (high/low) and gender (female/male) of the author as independent variables and message credibility score as the dependent variable to test for the hypothesized main effect of expertise (H1), as well as to answer ERQ2 and ERQ3. The two-way ANOVA showed neither a main effect of author expertise, *F*(1, 178) = 0.24, *p* = 0.626, nor a main effect of gender, *F*(1, 178) = 0.19, *p* = 0.662, or an interaction between the factors, *F*(1, 178) = 3.39, *p* = 0.067. Therefore, H1 was not supported. We also found no differences regarding ERQ2 and ERQ3. The credibility values for expertise and gender are depicted in Fig. 2.

To examine whether participants’ own gender influenced credibility ratings (ERQ1), an additional three-way ANOVA with participant gender as an additional between-groups factor was conducted. None of the main effects, *F*s < 0.92, *p*s > 0.434, neither of the two-way interactions, *F*s < 3.23, *p*s > 0.074, nor the three-way interaction, *F*(1, 172) = 0.42, *p* = 0.520, reached significance.

### Discussion

Study 2 was conducted to evaluate whether the effects observed in Study 1 would generalize to a more heterogeneous sample. For the gender of the author and the gender of the participants, we found again no significant effect. This is consistent with Study 1 and strengthens the conclusion that author and participant gender do not influence participants’ credibility perception under our design conditions. Thus, neither author nor participant gender were further investigated in the subsequent study.

The main effect of the authors’ expertise could not be replicated. Whereas Study 1 revealed a significant main effect of author expertise on perceived message credibility, Study 2 showed no such effect in the sample with larger demographic variability. This suggests a dependence of the effect observed in Study 1 on characteristics of the student sample. However, expertise is widely regarded as an established indicator of perceived credibility across various samples in the literature^24–28^, wherefore this explanation seems unlikely. A closer look reveals the study material as a plausible source for the inconsistent findings: While the low-expertise condition introduced the alleged author as being new to the topic at hand, they were still ascribed previous real-life experience and personal interest.

Expertise is not a homogeneous construct, but can encompass various forms of knowledge, skills, and abilities all perceived as expertise in real-life communication practice^29,46^. Specifically, it can be differentiated based on what grounds people are deemed to be experts: Weinstein^47^ differentiates between epistemic expertise, which is based on theoretical knowledge, and performative expertise, which is based on practical skills. Similarly, Wagemans^48^ also proposes a dichotomy between expert opinion based on professional knowledge acquired through professional training and experiential knowledge gained through personal experience. Following this breakdown of the concept of expertise, the author descriptions used in Study 1 and 2 possibly entailed an unintended dichotomy between professional academic expertise, and experience-based expertise. As different types of expertise may affect credibility perceptions to differing extents, this blend in our study material might have led to inconsistent results.

## Study 3

Therefore, we investigated whether there were differences in the effect of expertise on credibility judgments of science communication texts based on the type of expertise. This provides an important conceptual contribution to expertise research and may disentangle the conflicting results of Study 1 and 2. Previous research on source expertise often combined these expertise indicators^19^. However, this is not reflective of the current digital climate anymore, which has not only lowered the threshold to consume a vast amount of unfiltered information but has also opened the opportunity to provide information to a large audience independent of credentials. This, in turn, has led to a reexamination of standards for why authority in a topic is ascribed and whether other pathways than traditional academic education can lead to authority and expertise in a certain context^9,48^. Nevertheless, experimental research systematically differentiating different forms of expertise and their impact on perceived message credibility in science communication is still sparse.

Building on the differing effects of normatively strong vs normatively weak evidence on persuasion^49^, Burgers et al.^50^ empirically demonstrated the importance of this distinction. Their study found that the perceived expertise of technical experts (based on systematic professional knowledge) was significantly higher than that of experiential experts (based on practical experience), even though the perceived trustworthiness was roughly the same. In addition, the statements made by authors with professional knowledge were significantly more persuasive than those made by authors with experiential knowledge. Ferreira and Wingrove^51^ made a similar distinction between expert training and expert experience, showing that both factors had an independent impact on perceived credibility of forensic experts. We deduced that there might have been similar effects of the two forms of author expertise on perceived message credibility in our studies.

The author descriptions in Studies 1 and 2 contained professional academic as well as personal experiential expertise as described in Wagemans^48^ and Burgers et al.^50^. Academic expertise was based on formalized knowledge, academic training, and institutional recognition. Personal expertise was based on practical knowledge acquired through direct engagement with the topic. In Study 3, we explicitly differentiated between the two types of expertise and investigated whether there are differences in credibility judgments. We formulated the following hypotheses:

- H3: Main effect of academic expertise: A scientific text is perceived as more credible when allegedly written by an author with high academic expertise compared to an author with no academic expertise.
- H4: Main effect of personal expertise: A scientific text is perceived as more credible when allegedly written by an author with high personal expertise compared to an author with no personal expertise.
- H5: Combined effect: A scientific text is perceived as significantly more credible when allegedly written by an author with both forms of expertise (high academic and high personal expertise) compared to authors with only one form of expertise (either academic or personal).

Furthermore, we investigated in two exploratory research questions whether there is an interaction effect between the alleged academic expertise and personal expertise of an author of a scientific text (ERQ4), as well as whether there is a difference in the perception of credibility of scientific texts allegedly written by an author with exclusively academic expertise compared to an author with exclusively personal expertise (ERQ5).

### Methods

#### Participants

Study 3 was conducted as an online experiment using SoSci Survey (www.soscisurvey.de; Version 3.6.10)^44^. It contained a 2 × 2 between-groups design with academic expertise (high vs. none) and personal expertise (high vs. none) of the alleged author of a science communication text being varied.

Based on an a priori power analysis via G*Power (version 3.1.9.7), we intended to recruit a minimum of 222 participants to achieve 80% power for detecting a small effect (f^2^ = 0.05, α = 0.05) with a linear multiple regression (fixed model, R^2^ increase). Of the 268 participants who had completed the study, 62 had to be excluded due to the predefined exclusion criteria. Like in Studies 1 and 2, participants were excluded based on high prior knowledge in the topic (indicating 6 or 7 on a 7-point Likert scale) or a failed attention check. Participants were recruited via Prolific (www.prolific.com), an online participant recruitment platform. They received monetary compensation of 1,20€ for completing the study. They had to confirm that they were at least 16 years of age and had at least a C1 level of proficiency in German. For further demographic information see Table 1.

#### Procedure

Participants were informed that the goal of the study was to investigate the perception of scientific texts. After completing the informed consent, the participants were instructed to first read one of four descriptions of an author of a scientific text and then the text itself. They had a minimal reading time of 15 s for the author description and minimally 60 s for the scientific text. Participants were distributed equally among the four conditions (*n*_dual exp._ = 46, *n*_acad. exp._ = 48, *n*_pers. exp._ = 51, *n*_no exp.=_61). On the following page, the manipulation check was conducted using two questions regarding the degree of academic and personal expertise of the author in the subject area. Then the participants were presented with the same science communication text used in Studies 1 and 2. Afterwards, the participants indicated their perceived credibility of the text. They also performed the attention check and were asked to indicate their prior knowledge about natural disaster preparedness. The study concluded with demographic questions.

#### Materials and measures

Participants were presented with author descriptions reflecting four expertise conditions: (1) dual expertise (high academic and high personal expertise), (2) high academic expertise only, (3) high personal expertise only, and (4) no expertise. The descriptions were based on those of Study 1 and 2 but adapted to more concisely separate the types of expertise in the different conditions. Academic expertise was signaled by a doctorate, an extensive academic career, and international consulting experience. Personal expertise was conveyed through firsthand experience with a major flood and substantial practical engagement in flood prevention. In the no-expertise condition, the author was described as a newcomer to the topic with minimal prior involvement. We created the descriptions using ChatGPT-4 (OpenAI; Version 4.0)^45^. The stimuli are provided in the Supplementary Material (S4). The same science communication text was used as in Studies 1 and 2.

Message credibility was measured using the same 11-item scale as in Studies 1 and 2 (α = 0.95).

As a manipulation check, the participants independently answered the two questions “How much academic experience do you think the author has in the subject area?" and "How much personal experience do you think the author has in the subject area?” on 7-point Likert scales (1 = “no experience” and 7 = “extensive experience”). We specifically asked participants for the authors’ “experience” (not “expertise”) to avoid confusion over unfamiliarity with the phrase “personal expertise”.

The attention check consisted of the question “What is the gender of the author of the scientific text?” with the options: “female”, “male”, or “I don’t remember”. Since we used a common male first name and male pronouns in the author descriptions, participants who did not indicate “male” were excluded from the analysis.

To measure prior knowledge, we asked the same question as in the previous studies, also answered on a 7-point Likert scale. However, the labeling of the ends was switched to 1 = “not at all” and 7 = “fully applies”. Participants indicating 6 or 7 on the scale were, again, excluded from the analysis.

### Results

The statistical analysis was conducted using R (Version 4.3.0)^52^. The successful manipulation of both the academic and personal expertise was confirmed using two Welch’s t-tests. For academic expertise, *t*(200.13) = 21.56, *p* < 0.001, *d* = 3.02, the conditions with high academic expertise were regarded significantly higher in academic experience (*M* = 5.85, *SD* = 1.27) than the conditions without academic expertise (*M* = 1.96, *SD* = 1.32). For personal expertise, *t*(160.56) = 16.92, *p* < 0.001, *d* = -2.29, the conditions with high personal expertise were regarded significantly higher in personal experience (*M* = 6.27, *SD* = 0.90) than the conditions without personal expertise (*M* = 2.92, *SD* = 1.84).

**Fig. 3:**
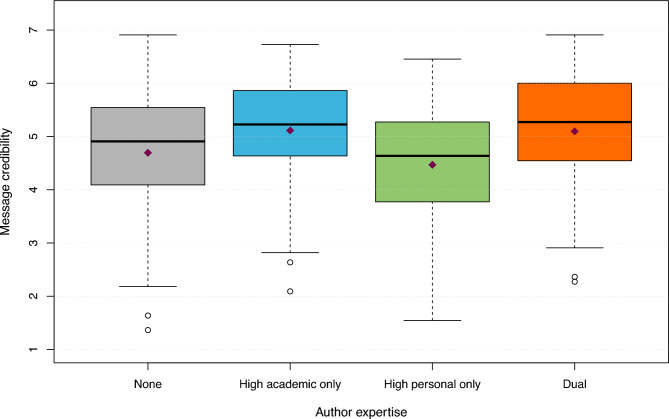
Boxplot of the perceived message credibility values for the four experimental conditions in Study 3. Mean credibility values are indicated with a lilac diamond.

To understand whether there was a main effect of academic expertise (H3), a main effect of personal expertise (H4), or an interaction between the two (ERQ4), a linear model was used. The overall model was significant, *F*(3, 202) = 3.65, *p* = 0.013, *R*^2^ = 0.05. Academic expertise was a significant positive predictor of perceived message credibility (β = 0.52, *p* = 0.002), whereas personal expertise (β = -0.12, *p* = 0.459) and the interaction (β = 0.21, *p* = 0.524) were not significant. This supports H3, while H4 could not be supported and ERQ4 was negated. The credibility values of all four conditions are depicted in Fig. 3.

To investigate whether there was a combined effect of the types of expertise compared to singular expertise (H5) and a difference between the exclusively presented types of expertise (ERQ5), we fitted a linear model with contrast coding (CH5, CE5, R1). Contrast coding was applied as follows: CH5 = [0, -0.5, -0.5, 1], CE5 = [0, -0.5, 0.5, 0], R1 = [-0.75, 0.25, 0.25, 0.25], with^1, 2, 3, 4^ referring to the experimental conditions 1) no expertise, 2) no academic / high personal expertise, 3) high academic / no personal expertise, and 4) high academic / high personal expertise. The overall model was significant, *F*(3, 202) = 3.65, *p* = 0.013, *R*^2^ = 0.05. The contrast CH5, representing the contrast between dual and the average of the singular types of expertise (H5), was not significant (β = 0.20, *p* = 0.144). Since we had assumed that a scientific text is perceived as significantly more credible when allegedly written by an author with both forms of expertise (high academic and high personal expertise) compared to authors with only one form of expertise (either academic or personal), H5 could, therefore, not be supported. Contrast CE5, however, which solely compared exclusively academic with exclusively personal expertise (ERQ5), was a significant positive predictor of credibility (β = 0.64, *p* = 0.007), answering ERQ5 affirmatively. Therefore, there was a difference in the perception of credibility of scientific texts allegedly written by an author with exclusively academic expertise compared to an author with exclusively personal expertise with academic expertise, due to the direction of the contrast, being a positive predictor. The residual contrast R1, which compared all three expertise conditions with the control condition of no expertise, was not significant (β = 0.20, *p* = 0.266).

### Discussion

Study 3 showed a significant main effect of academic expertise, indicating higher perceived credibility for texts written by sources with high academic expertise than sources with no academic expertise. Academic-only sources were also rated more credible than personal-only sources when only one type of expertise was present. The significance of academic expertise is in accordance with literature indicating effects of expertise on credibility perception^24,28^. Furthermore, it is partially in accordance with the findings by Burgers et al.^50^ and Ferreira and Wingrove^51^, who showed increased persuasiveness of people with professional compared to personal expertise and independent effects of professional expertise on credibility assessments, respectively.

## General discussion

Correctly judging the credibility of science communication texts without proper expertise in the field is an essential skill for laypeople. Besides the content, other properties of the communication environment, like information about the author, can be used to approximate message credibility. Therefore, it is important to critically regard the impact of author features in general and expertise cues in particular on the subjective perception of messages in science communication.

In three experimental studies, we investigated whether the alleged expertise and gender of an author impact the perceived message credibility of a gender-neutral science communication text. In terms of author gender, Study 1 and 2 showed no significant effects of author gender on perceived message credibility. The participants’ own gender was also no moderator of the credibility judgments. We interpreted the results of Study 1 and 2 as supporting the current trend toward gender-independent credibility assessments of science communication^38–40^.

While Study 1 found an effect of author expertise on message credibility with alleged high author expertise leading to increased credibility ratings in a student sample, Study 2 failed to replicate this effect with a more diverse sample. While labelling the conditions in Studies 1 and 2 as high or low in expertise, the conditions comprised a mixture of academic and personal expertise. Strictly differentiating between the two concepts, we investigated their specific impact on message credibility in Study 3. We found that only academic, but not personal expertise had a significant effect on perceived message credibility. We also did not find additive or interactive effects of both types of expertise.

These results are only partially in accordance with the literature, which indicates independent effects for both academic and personal expertise^50,51^. In accordance with the Elaboration Likelihood Model^32,53^, we assume that the presented science communication text was judged based on heuristics activated by the author descriptions, which were more favorable to established cues like academic credentials and thereby may reduce processing effort for the academic expertise condition^34,54,55^. In contrast, personal expertise requires more elaborate processing due to its heterogeneity. Recipients must evaluate the relevance of individual experiences to scientific claims. This is a process that requires high motivation and cognitive resources^56^. This increases processing demands, which participants might not have invested. This reasoning is supported by the consideration that the text itself was probably not personally relevant to the participants and was thus processed with low effort, mostly relying on heuristic processing^54,57,58^.

The discrepancy could also be explained using the Unifying Framework of Credibility Assessment^33^. It is conceivable that when confronted with the author information, an initial assessment was made at the construct level of which heuristics can be activated by the information present. Although personal expertise could have been perceived as a peripheral source reference at the interaction level, it presumably did not align with the recipients’ science-specific understanding of credibility as academic expertise did. This could be particularly true for a complex, systemic issue such as climate change, which may transcend the limits of individual experience. For topics such as mental illness, personal expertise might be perceived as a more valuable credibility cue due to a more deeply felt sense of personal involvement. Therefore, while academic expertise may have activated appropriate heuristics, the non-standard personal expertise was unable to establish a comparable link between the perceived signal and a suitable credibility heuristic. Mere perception at the interaction level is, therefore, insufficient if no suitable heuristic exists that is compatible with the abstract credibility construct. The framework thus demonstrates how all three levels must interact for a cue to lead to a positive credibility judgment.

The symbolic power of academic titles probably also carries specific weight in relation to the results of Study 1. Academic titles represent institutional validation by the scientific system^59,60^ and the symbolic power of academic titles activates culturally embedded associations with authority^21,61^. Therefore, academic expertise specifically acts as an established signal of credibility through institutional legitimation and formalized knowledge structures^26,59,62^. In contrast, experience-based expertise represents an alternative form of knowledge legitimation through practical provenance^48^. The concept of a *recognition heuristic*^54^ suggests that the familiarity of academic titles can act as a signal of credibility. University students in particular are embedded in the academic realm and potentially used to accepting academic credentials as a sign of epistemic authority^63,64^. The incomplete separation of academic and personal expertise may have been processed more variably by the sample of Study 2, which might be more removed from academia. This could have potentially led to the non-significant findings in Study 2 for author expertise, while the student sample probably focused more on the academic credentials as a signal of epistemic authority, leading to the significant findings in Study 1. We attribute the reappearance of the significant main effect for author expertise in Study 3 to the more precise distinction between types of expertise in the material used for Study 3.

In summary, we observed that the effect of author expertise varied across studies: It was significant in Study 1 (student sample) and Study 3 (general sample), but not in Study 2 (general sample). We attribute this variability to students’ greater sensitivity to academic achievement, which may have offset the dilutive effects of the less clearly defined materials in Studies 1 and 2. In Study 3, where expertise was more clearly differentiated, the effect emerged independently of sample characteristics. However, the results have to be considered in the context of the study environment. The present studies used isolated online settings with controlled text presentations, whereas science communication often takes place in more complex, interactive media environments. Zimmermann et al.^28^, for example, demonstrated that on Instagram, political expertise significantly increased perceived credibility, while traditional source types had little influence. This highlights how much the context of the medium can influence expertise effects besides personal factors like processing motivation and capacity^32^. Moreover, the technicality of the topic might explain a preference for academic expertise over personal expertise, which might not have been present in more socially-oriented topics. Therefore, future studies should examine the robustness of the effects of different types of expertise in different digital media and communication environments and across various topics.

## Conclusion

Our findings support the notion that an author’s academic credentials provide a decisive advantage regarding the perceived credibility of a science communication text, while personal expertise does not add credibility in this context. The clear hierarchy between different forms of expertise confirms that not all expertise signals are equally effective. This clarifies previous assumptions about the effects of expertise on credibility perceptions and shows that the type of expertise, and not its presence in general, is crucial in science communication.

## Supplementary Information

Supplementary information.

## Data Availability

The datasets generated and analyzed during the current study are available in the Open Science Framework, https://osf.io/qjts4/overview?view_only=508b142e9ea54a08b9791056df455cf7
