# Supplementary material for: Authors’ academic but not personal expertise or gender affect message credibility in science communication
Source: Sci Rep. 2026 Jun 10;16:18000. doi: 10.1038/s41598-026-55148-x (PMC13253843; doi:10.1038/s41598-026-55148-x)
Supplement: Supplementary file 1 — Supplementary information. [file 41598_2026_55148_MOESM1_ESM.pdf]

Authors' academic but not personal expertise or gender affect message credibility in science communication

Annalena Ulsperger[1,*], Lara Pfannenschwarz[2], Clara Schetla[2], Karen Poletilo[2], Lina Strecker[2], Marlene Egner[2], Joachim Kimmerle[1,2]

[1]Leibniz Institut für Wissensmedien (IWM), Tübingen, Germany

[2]University of Tübingen, Tübingen, Germany

*a.ulsperger@iwm-tuebingen.de

Supplementary Material

S1

The following author descriptions were used in Studies 1 and 2:

High expertise, male author

[Original]

Herr Dr. Michael Weber hat sich durch seine sehr umfangreiche Forschung und praxisorientierte Studien im Bereich des naturbasierten Schutzes vor Naturkatastrophen hervorgetan. Seine Forschung ist die Grundlage von einigen weitreichenden Projekten und wurde bereits in mehreren renommierten Fachzeitschriften publiziert. Zudem berät er weltweit über Möglichkeiten, wie Ökosysteme selbst zur Minderung der Auswirkungen von Naturkatastrophen beitragen können. Im folgenden Text teilt er seine Ansicht, wie mit nachhaltigen und ökologischen Mitteln ein bestmöglicher Schutz vor Naturkatastrophen erzielt werden kann.

[English translation]

Dr. Michael Weber has distinguished himself through his extensive research and practice-oriented studies in the field of nature-based protection against natural disasters. His research forms the basis of several far-reaching projects and has already been published in a number of renowned journals. He also advises clients worldwide on ways in which ecosystems themselves can help mitigate the effects of natural disasters. In the following text, he shares his views on how sustainable and ecological means can be used to achieve the best possible protection against natural disasters.

Low expertise, male author

[Original]

Herr Michael Weber ist neu im Themenbereich des naturbasierten Schutzes vor Naturkatastrophen. Sein Wissen beruht hauptsächlich auf gesammelten Erfahrungen, als seine Heimatstadt von einem Jahrhunderthochwasser betroffen war. Obwohl er keine akademische Ausbildung in diesem Themenbereich hat, beschäftigt er sich mit praktischen Möglichkeiten, wie die Kräfte von Ökosystemen zu einer Minderung der Auswirkungen von Naturkatastrophen beitragen können. Im folgenden Text teilt er seine Ansichten, wie mit nachhaltigen und ökologischen Methoden ein bestmöglicher Schutz vor Naturkatastrophen erreicht werden kann.

[English translation]

Mr. Michael Weber is new to the field of nature-based protection against natural disasters. His knowledge is mainly based on experience gained when his hometown was hit by a once-in-a-century flood. Although he has no academic training in this field, he is interested in practical ways in which the forces of ecosystems can help mitigate the effects of natural disasters. In the following text, he shares his views on how sustainable and ecological methods can be used to achieve the best possible protection against natural disasters.

High expertise, female author

[Original]

Frau Dr. Michaela Weber hat sich durch ihre sehr umfangreiche Forschung und praxisorientierte Studien im Bereich des naturbasierten Schutzes vor Naturkatastrophen hervorgetan. Ihre Forschung ist die Grundlage von einigen weitreichenden Projekten und wurde bereits in mehreren renommierten Fachzeitschriften publiziert. Zudem berät sie weltweit über Möglichkeiten, wie Ökosysteme selbst zur Minderung der Auswirkungen von Naturkatastrophen beitragen können. Im folgenden Text teilt sie ihre Ansicht, wie mit nachhaltigen und ökologischen Mitteln ein bestmöglicher Schutz vor Naturkatastrophen erzielt werden kann.

[English translation]

Dr. Michaela Weber has distinguished herself through her extensive research and practice-oriented studies in the field of nature-based protection against natural disasters. Her research forms the basis of several far-reaching projects and has already been published in a number of renowned journals. She also advises clients worldwide on ways in which ecosystems themselves can help mitigate the effects of natural disasters. In the following text, she shares her views on how sustainable and ecological means can be used to achieve the best possible protection against natural disasters.

Low expertise, female author

[Original]

Frau Michaela Weber ist neu im Themenbereich des naturbasierten Schutzes vor Naturkatastrophen. Ihr Wissen beruht hauptsächlich auf gesammelten Erfahrungen, als ihre Heimatstadt von einem Jahrhunderthochwasser betroffen war. Obwohl sie keine akademische Ausbildung in diesem Themenbereich hat, beschäftigt sie sich mit praktischen Möglichkeiten, wie die Kräfte von Ökosystemen zu einer Minderung der Auswirkungen von Naturkatastrophen beitragen können. Im folgenden Text teilt sie ihre Ansichten, wie mit nachhaltigen und ökologischen Methoden ein bestmöglicher Schutz vor Naturkatastrophen erreicht werden kann.

[English translation]

Michaela Weber is new to the field of nature-based protection against natural disasters. Her knowledge is mainly based on the experience she gained when her hometown was hit by a once-in-a-century flood. Although she has no academic training in this field, she is interested in practical ways in which ecosystems can help mitigate the effects of natural disasters. In the following text, she shares her views on how sustainable and ecological methods can be used to achieve the best possible protection against natural disasters.

S2

The following science communication text about natural disaster preparedness was used in all conditions Studies 1-3. Participants had to judge the message credibility of said text.

[Original]

Natürliche Ökosysteme zur Naturkatastrophenvorsorge

Die Nutzung von Ökosystemen als natürlicher Schutz vor Naturkatastrophen ist ein Ansatz, der im Bereich des Katastrophenschutzes zunehmend an Bedeutung gewinnt und zusätzliche externe Maßnahmen überflüssig werden lässt. Diese Methode setzt auf die natürliche Schutzfunktion, die bestimmte Ökosysteme wie Mangrovenwälder, Korallenriffe oder Feuchtgebiete bieten, um die Auswirkungen von Naturkatastrophen zu mildern. Mangrovenwälder entlang der Küste, zum Beispiel, können Sturmfluten und Wellen abmildern, da ihre Wurzel bis zu 70% der Wellenenergie abfangen - ein natürlicher Schutzmechanismus, der technische Alternativen oft übertrifft. Ebenso schützen Seegraswiesen an Küstengebiete vor Überschwemmungen bei Starkregen, indem bereits ein Hektar bis zu 80.000 Liter Wasser pro Stunde filtern kann.

Die Aufrechterhaltung solcher Ökosysteme ermöglicht es, deren natürlichen Schutzmechanismus zu nutzen und so die Gefährdung von Menschen und Infrastruktur zu reduzieren. Durch die gezielte Pflege und den Schutz solcher Gebiete entsteht ein widerstandsfähiges Schutzsystem, das sich selbst erhält und an veränderte Umweltbedingungen anpasst. Diese natürliche Schutzstrategie erübrigt oft den Einsatz kostenintensiver Bauprojekte und macht künstliche Barrieren, wie Dämme oder Talsperren, entbehrlich.

Ein rein natürlicher Schutz durch Ökosysteme ist demnach so effektiv, dass zusätzliche Maßnahmen kaum erforderlich sind, um vor Naturkatastrophen zu schützen. Während künstliche Schutzanlagen regelmäßig überprüft, repariert und manchmal sogar erneuert werden müssen, übernehmen natürliche Barrieren diese Aufgabe eigenständig und regenerieren sich kontinuierlich. Die sogenannten Salzmarschen, bestehend aus salztoleranten Pflanzen an Küsten und Flussmündungen, wachsen beispielsweise stetig mit dem Meeresspiegel mit. Dies funktioniert, indem sie bei steigendem Wasserspiegel zusätzliches Sediment ablagern, wodurch sie ihre Schutzfunktion gegen Überschwemmungen und Küstenerosion beibehalten oder sogar verstärken.

[English translation]

Natural ecosystems for natural disaster preparedness

The use of ecosystems as natural protection against natural disasters is an approach that is becoming increasingly important in the field of disaster control and makes additional external measures unnecessary. This method relies on the natural protective function offered by certain ecosystems such as mangrove forests, coral reefs, or wetlands to mitigate the effects of natural disasters. Mangrove forests along the coast, for example, can mitigate storm surges and waves, as their roots absorb up to 70% of wave energy – a natural protection mechanism that often surpasses technical alternatives. Similarly, seagrass beds protect coastal areas from flooding

during heavy rainfall, with just one hectare capable of filtering up to 80,000 liters of water per hour.

Maintaining such ecosystems makes it possible to utilize their natural protective mechanisms, thereby reducing the risk to people and infrastructure. The targeted maintenance and protection of such areas creates a resilient protection system that is self-sustaining and adapts to changing environmental conditions. This natural protection strategy often eliminates the need for costly construction projects and makes artificial barriers such as dams or reservoirs unnecessary.

Purely natural protection provided by ecosystems is so effective that additional measures are hardly necessary to protect against natural disasters. While artificial protective structures must be regularly inspected, repaired, and sometimes even replaced, natural barriers perform this task independently and regenerate continuously. Salt marshes, for example, which consist of salt-tolerant plants on coasts and river mouths, grow steadily with sea level. This works by depositing additional sediment as the water level rises, thereby maintaining or even enhancing their protective function against flooding and coastal erosion.

S3

The following scale was used to measure perceived message credibility in Studies 1-3.

Dimension	Item		Source
	Original	***English translation***	*Adapted from...*
Trustworthiness	Ich halte die Aussagen im Text für vertrauenswürdig	I consider the statements in the text to be trustworthy.	Klebolte (2019)
	Ich glaube den Textaussagen, ohne weitere Recherchen vorzunehmen.	I believe the statements in the text without conducting further research.	
	Auch ohne weiteres Hintergrundwissen sind die Aussagen für mich glaubwürdig.	Even without further background knowledge, I find the statements credible.	
	Bevor ich diese Aussagen weitererzähle, würde ich eine zweite Meinung einholen.	Before I pass on these statements, I would seek a second opinion.	
Truth	Der Text gibt die Dinge so wieder, wie sie sind.	The text reflects things as they are.	Mehlis (2014)
	Der Text informiert über Fakten und Tatsachen.	The text provides information about facts and matters of fact.	
	Der Text bringt die ungeschminkte Wahrheit.	The text tells the unvarnished truth.	
Agreement	Ich würde einem Freund in zustimmender Weise von diesem Text berichten.	I would tell a friend about this text in an approving manner.	Klebolte (2019)
	Bei einer Diskussion würde ich den Inhalt des Textes befürworten.	In a discussion, I would support the content of the text.	
	Ich stimme den Inhalten des Textes zu.	I agree with the contents of the text.	Added by the authors
	Wenn ich gefragt werde, würde ich mich positiv zur im Text vertreten Meinung äußern.	If asked, I would express a positive opinion on the view expressed in the text.	

Note: *Item four in the trustworthiness-dimension is inverse coded.*

S4

The following author descriptions were used in Study 3:

No expertise

[Original]

Michael Weber ist noch neu im Themenbereich des naturbasierten Schutzes vor Naturkatastrophen. Bisher hat er weder akademische noch persönliche Erfahrungen in dem Feld Naturkatastrophenschutz gesammelt. Er interessiert sich jedoch für das Thema und hat begonnen, sich in seiner Freizeit mit Konzepten auseinanderzusetzen, wie die Kräfte von Ökosystemen zu einer Minderung der Auswirkungen von Naturkatastrophen beitragen können. Im folgenden Text teilt er seine Ansichten, wie mit nachhaltigen und ökologischen Methoden ein bestmöglicher Schutz vor Naturkatastrophen erzielt werden kann.

[English translation]

Michael Weber is still new to the field of nature-based protection against natural disasters. To date, he has no academic or personal experience in the field of natural disaster protection. However, he is interested in the topic and has begun to explore concepts in his spare time on how the forces of ecosystems can help mitigate the effects of natural disasters. In the following text, he shares his views on how sustainable and ecological methods can be used to achieve the best possible protection against natural disasters.

High academic / low personal expertise

[Original]

Dr. Michael Weber hat sich durch seine umfangreiche Forschung im Bereich des naturbasierten Schutzes vor Naturkatastrophen hervorgetan. Seine Forschung ist die Grundlage einiger weitreichender Projekten und wurde in mehreren renommierten Fachzeitschriften publiziert. Obwohl er keine persönlichen Erfahrungen in diesem Themenbereich hat, berät er weltweit über Möglichkeiten, wie Ökosysteme zur Minderung der Auswirkungen von Naturkatastrophen beitragen können. Im folgenden Text teilt er seine Ansicht, wie mit nachhaltigen und ökologischen Mitteln ein bestmöglicher Schutz vor Naturkatastrophen erzielt werden kann.

[English translation]

Dr. Michael Weber has distinguished himself through his extensive research in the field of nature-based protection against natural disasters. His research forms the basis of several far-reaching projects and has been published in a number of renowned journals. Although he has no personal experience in this field, he advises clients worldwide on how ecosystems can help mitigate the effects of natural disasters. In the following text, he shares his views on how sustainable and ecological means can be used to achieve the best possible protection against natural disasters.

Low academic / high personal expertise

[Original]

Michael Weber setzt sich viel mit dem naturbasierten Schutz vor Naturkatastrophen auseinander. Sein Wissen beruht hauptsächlich auf gesammelten Erfahrungen, als seine Heimatstadt von einem Jahrhunderthochwasser betroffen war. Obwohl er keine akademische Erfahrung in diesem Themenbereich hat, beschäftigt er sich praktisch umfassend damit, wie die Kräfte von Ökosystemen zu einer Minderung der Auswirkungen von Naturkatastrophen beitragen können. Im folgenden Text teilt er seine Ansichten, wie mit nachhaltigen und ökologischen Methoden ein bestmöglicher Schutz vor Naturkatastrophen erzielt werden kann.

[English translation]

Michael Weber is very interested in nature-based protection against natural disasters. His knowledge is mainly based on the experience he gained when his hometown was hit by a once-in-a-century flood. Although he has no academic background in this field, he has extensive practical experience in how the forces of ecosystems can help mitigate the effects of natural disasters. In the following text, he shares his views on how sustainable and ecological methods can be used to achieve the best possible protection against natural disasters.

Dual expertise

[Original]

Dr. Michael Weber hat sich durch seine umfangreiche Forschung im Bereich des naturbasierten Schutzes vor Naturkatastrophen hervorgetan. Seine Forschung ist die Grundlage einiger weitreichenden Projekten und wurde in mehreren renommierten Fachzeitschriften publiziert. Auf Basis seiner akademischen Erfahrung berät er weltweit über Möglichkeiten, wie Ökosysteme zur Minderung der Auswirkungen von Naturkatastrophen beitragen können. Nachdem er erlebte, wie seine Heimatstadt von einem Jahrhunderthochwasser betroffen war, fließen diese persönlichen Erfahrungen zudem stark in seine Arbeit ein. Im folgenden Text teilt er seine Ansicht, wie mit nachhaltigen und ökologischen Mitteln ein bestmöglicher Schutz vor Naturkatastrophen erzielt werden kann.

[English translation]

Dr. Michael Weber has distinguished himself through his extensive research in the field of nature-based protection against natural disasters. His research forms the basis of several far-reaching projects and has been published in a number of renowned journals. Drawing on his academic experience, he advises clients worldwide on how ecosystems can help mitigate the effects of natural disasters. Having witnessed his hometown being hit by a once-in-a-century flood, his personal experiences also play a major role in his work. In the following text, he shares his views on how sustainable and ecological measures can be used to achieve the best possible protection against natural disasters.
